# Object Categorization Capability of Psychological Potential Field in Perceptual Assessment Using Line-Drawing Images

**DOI:** 10.3390/jimaging8040090

**Published:** 2022-03-26

**Authors:** Naoyuki Awano, Yuki Hayashi

**Affiliations:** 1 Faculty of Business, Kokushikan University, 4-28-1 Setagaya, Setagaya-ku, Tokyo 154-8515, Japan; 2 Graduate School of Humanities and Sustainable System Sciences, Osaka Prefecture University, 1-1 Gakuen-cho, Naka-ku, Sakai 599-8531, Japan; hayashi@kis.osakafu-u.ac.jp

**Keywords:** fixation map, psychological potential field, line drawing, eye tracking, perceptual assessment

## Abstract

Affective/cognitive engineering investigations typically require the quantitative assessment of object perception. Recent research has suggested that certain perceptions of object categorization can be derived from human eye fixation and that color images and line drawings induce similar neural activities. Line drawings contain less information than color images; therefore, line drawings are expected to simplify the investigations of object perception. The psychological potential field (PPF), which is a psychological feature, is an image feature of line drawings. On the basis of the PPF, the possibility that the general human perception of object categorization can be assessed from the similarity to fixation maps (FMs) generated from human eye fixations has been reported. However, this may be due to chance because image features other than the PPF have not been compared with FMs. This study examines the potential and effectiveness of the PPF by comparing its performance with that of other image features in terms of the similarity to FMs. The results show that the PPF shows the ideal performance for assessing the perception of object categorization. In particular, the PPF effectively distinguishes between animal and nonanimal targets; however, real-time assessment is difficult.

## 1. Introduction

Affective/cognitive engineering investigations generally require the quantitative assessment of the human perception of objects. Electroencephalogram records, which measure electrical activity in the brain, are useful for assessing diverse perceptions [1]. Human eye tracking is also important for measuring human perceptions of vision. Eye trackers directly record eye movements such as fixations and saccades [2] which have been widely used in eye movement analysis. However, they only directly represent viewed locations; therefore, perceptual trends must be extracted according to objectives.

Recent studies have reported that certain perceptions of object categorization may be derived from eye fixations and that color images and line drawings generate similar neural activities [3,4]. These findings have simplified object perception investigations because the examination of visual perceptions in color images and associated eye fixations are complex. Furthermore, the perceptual quantities of object categorization may be assessed from eye fixations and a line-drawing image feature, i.e., the psychological potential field (PPF) [5]. Specifically, the similarity values between the PPF [6] and fixation maps (FMs) for visualizing eye fixations mimic actual human perceptions. This could be a reasonable result from the perspective of analyzing viewed images and eye fixations.

**Figure 1:**
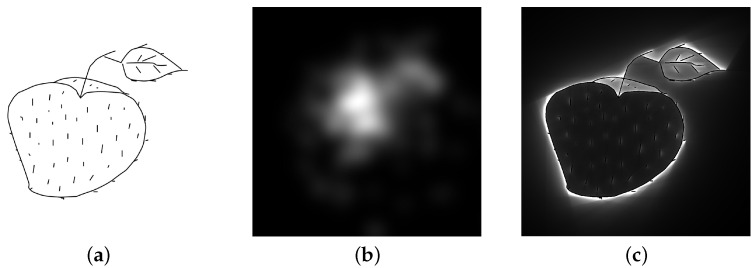
Example of the fixation map [16] and the psychological potential field (PPF): (**a**) stimulus image; (**b**) fixation map; and (**c**) psychological potential field.

The PPF was discovered using a light threshold method to investigate the psychological impacts of shape contours on vision [7], and it has been applied in physiological and psychological investigations [8,9]. The PPF is a potential field of psychological intensities around shapes, and it is similar to an electrostatic field. It can be computed from shape contours under the conditions that an object (foreground) is black and the ground (background) is white, as shown in Figure 1. In physiology, the PPF is a visualization of a phenomenon that occurs between the retina and brain, and it is suggested as a meta function before or during perception [10]. However, according to Gestalt psychology, perceptual organization, which is composed of grouping and segregation processes [11], is applied when an object is viewed. The PPF mechanism occurs before the grouping and segregation processes; hence, it can be regarded as a phenomenon that occurs between sensation and perception. Furthermore, because a continuous low-spatial-frequency field is formed around shapes, as shown in Figure 1c, the PPF might be relevant to gist perception, indicating that low-spatial-frequency components are important [12]. However, the effects of the PPF on perception and recognition are unclear. Nevertheless, a few studies applied the PPF to human Kansei evaluation, such as lettering design [13], arch bridge design [14], and female hairstyle [15], and demonstrated its effectiveness. In addition, a previous study [5] indicated that the PPF can be applied to object categorization. Considering these results, further investigation of the PPF may contribute to clarifying the multiple mechanisms of perception and cognition.

As mentioned above, a previous study [5] compared the PPF and FMs and reported that their similarity may be used to assess the visual perceptions of object categorization. Currently, the computational PPF theory was only established for two-dimensional binary images such as line drawings [17,18]. That is, the PPF is a figure-dependent distributed static image feature. However, there are several other figure-dependent distributed static features for line-drawing images. For example, lines are the most basic elements of line drawings and the distance field [19] is a well-known conventional feature. However, a previous study of the similarities between the PPF and FMs attempted to elucidate the distinction between the PPF and FMs rather than assessing visual perceptions of object categorization. Hence, the effectiveness of the PPF must be clarified when the similarity between the PPF and FMs is used to assess the visual perceptions of object categorization. This is because PPF usage in object categorization has never been validated and the similarity between other distributed static image features and FMs may reproduce similar or better trends compared to the PPF.

This study compares the similarity between FMs and four representative image features of binary line drawings, including the PPF, to determine which image feature shows the best performance in distinguishing between three fundamental object categories (animate objects, inanimate objects, and meaningless shapes), from the viewpoint of human perception. This provides evidence for the adequacy of the PPF usage in object categorization. Furthermore, this also indicates whether the PPF contains perceptual effects that are relevant to sensation and recognition processes.

## 2. Fixation Maps

### 2.1. Eye Tracking

Numerous studies have adopted eye tracking for various objectives because eyes can provide important perceptual information on human vision. For example, eye tracking has been used in studies on mental workload monitoring [20], health assessment [21,22], user interfaces [23], and learning methods for metacognitive skill training [24]. Eye tracking information includes eye saccades and fixations to stimuli as the primary types of eye movements. An eye saccade is defined as the rapid movement of the fovea from one point of interest to another, and it represents eye movement transitions. Eye fixations are defined as the periods during which the eye is aligned with a target, and they represent the viewed locations of observers [25]. One or both of these types of information are used depending on objectives.

### 2.2. Fixation Map Generation

Eye trackers can obtain numerous points as fixation locations, and FMs are images visualizing fixations based on those points. A three-step approach is used to construct FMs by considering a specific range of foveal vision and fixation locations [26]. First, the fixation points are rendered and accumulated on a preprepared blank image using a scaling function based on the foveal vision and the distance between the eyes and a target. A Gaussian function is commonly used as the scaling function, and the value of σ is selected depending on the situation [27]. Second, the fixation intensities of the images are normalized. Short-duration fixations are typically neglected, whereas long-duration fixations on the objects of interest are highlighted. Finally, grayscale or rainbow gradient colorization is used for visualization. Figure 1 shows an example of an FM in which white indicates high fixation intensities (frequently viewed locations) and black indicates low fixation intensities (unviewed locations).

Furthermore, predictive maps have been studied because eye-tracking devices are expensive. Several recent studies have attempted to generate FMs using machine learning as a saliency map [28,29]. In addition, studies have captured eye movements using a webcam instead of an eye tracker [30] or substituting mouse clicks for eye fixations [31,32].

## 3. Image Features and Similarity Metric

### 3.1. Stimulus Images and Experimental Setups

**Figure 2:**
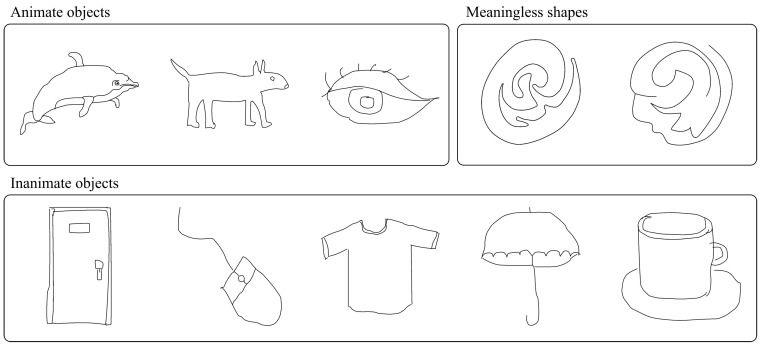
Stimulus images: animate objects (dolphin, dog, and eye), inanimate objects (door, mouse, T-shirt, umbrella, and cup), and meaningless shapes (MS1 and MS2).

We used ten images of simple line-drawing objects as stimuli to reduce the burden on the experimental participants, as shown in Figure 2. They were binary images consisting of pixels with black foreground and white background values with a resolution of 1080×1080. They were classified into three categories: three animate objects (dolphin, dog, and eye), five inanimate objects (door, mouse, T-shirt, umbrella, and cup), and two meaningless shapes (MS1 and MS2). The images of the animate and inanimate objects were selected from the MIT/Tübingen Saliency Benchmark datasets [33]. The images of the meaningless shapes were selected from a previous study on meaningless shapes not associated with common objects that multiple observers could not identify [34].

**Figure 3:**
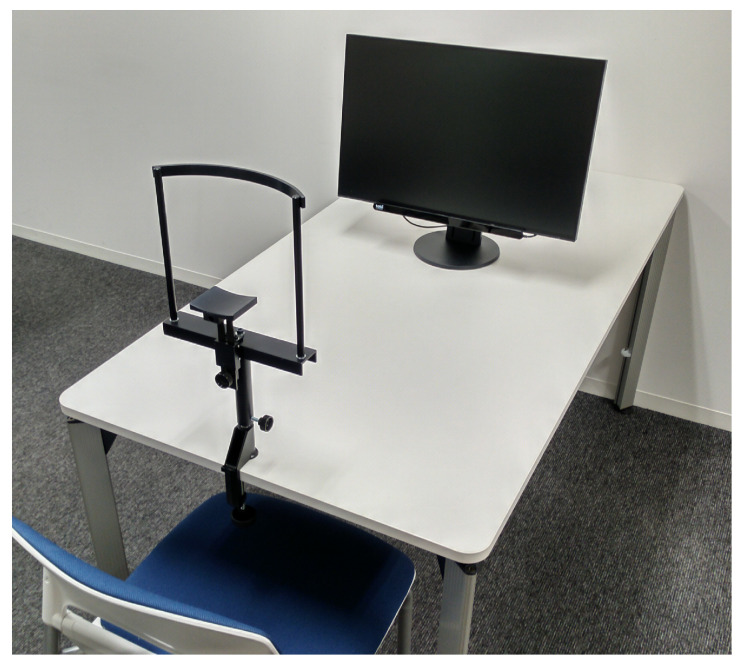
Experimental setup. The participants were sat in front of the display. We explained the experiment and obtained informed consent from each participant. Each participant’s chin was placed on the static chin rest. The experiment was conducted after calibrating the eye tracker according to each participant.

Figure 3 shows the experimental setup. We used a 24.1${}^{\prime \prime }$ display, Tobii Eye Tracker 4C, and a static chin rest for fixing the head. Each image was rendered at the center of the full-screen display with a gray background. The distance between a participant’s eyes and the center of the display was 70 cm. The visual angle calculated based on the length of the displayed image on the display was 23.5°.

The experimental participants were five males and five females with a mean age of 20 years, and they were recruited from university students. All participants were seated in front of the display and requested to freely view each stimulus for 30 s. We did not inform the participants about the object category to obtain neutral eye responses, and they freely viewed the stimuli without judging the object category. The order in which the stimuli were displayed was randomly determined for each participant.

The FMs of the stimuli were generated by the method described in Section 2.2 from the fixation points obtained by the above experiment. FMs of binary line drawings were gender-independent in a previous study [5]; thus, the FMs in this study were generated from all ten participants.

### 3.2. Image Features and Similarity Metric

The values of the image pixels were set as 0.0–1.0 (0.0 for black pixels and 1.0 for white pixels).

#### 3.2.1. Fundamental Similarity Metric

Location-based and distribution-based metrics have been proposed as methods for determining the similarity of FMs and other images. Location-based metrics such as the area under a receiver operating characteristic curve [35], normalized scanpath saliency (NSS) [36] and information gain [37] calculate the similarities at discrete fixation locations. In contrast, distribution-based metrics such as Pearson’s correlation coefficient (CC) and similarity [38] calculate the image similarities as continuous distributions. Currently, NSS is the fairest comparison for location-based metrics and the CC for distribution-based metrics [39]. In this study, we compared FMs and the four image features, which were distributed static features; thus, the CC was adopted as the similarity. However, as there were different details in each image feature, CCs with different preconditions based on the image features were adopted. This is explained in the following sections.

#### 3.2.2. Binary Feature

Foreground lines are the basic image features in binary line drawings. Therefore, as the binary feature (BIN), we set the pixels on the lines as 1.0 and all background pixels as 0.0 to match the FM.

The similarity of the BIN to the FM was estimated using the CC. The CC evaluated the degree to which the participants viewed the lines of each stimulus.

#### 3.2.3. Reciprocal Distance Field

The distance field [19] is a well-known binary image feature that stores the distance between each background pixel and its nearest foreground pixel. Low values are assigned to the pixels that are close to the line pixels. We constructed a reciprocal distance field (RDF) from the reciprocals of each value in the distance field.

The similarity of the RDF to the FM was evaluated using the CC. However, the pixel values of the lines in the distance field were zero; thus, their reciprocals could not be determined. We excluded the values of the line pixels from the evaluation of the CC. The CC evaluated the degree to which the surrounding areas of the lines were viewed by the participants.

#### 3.2.4. BIN + RDF

The BIN-FM similarity evaluated the degree to which the lines were viewed but excluded surrounding areas. In contrast, the RDF-FM similarity evaluated the degree to which the surrounding areas were observed but excluded the lines. The BIN and RDF were combined to evaluate the lines and their surrounding areas. We set the pixel values of the lines in the RDF to 1.0.

Similarly to the BIN-FM similarity calculation, the CC evaluated the degree to which the lines and their surrounding areas were viewed.

#### 3.2.5. Psychological Potential Field

**Figure 4:**
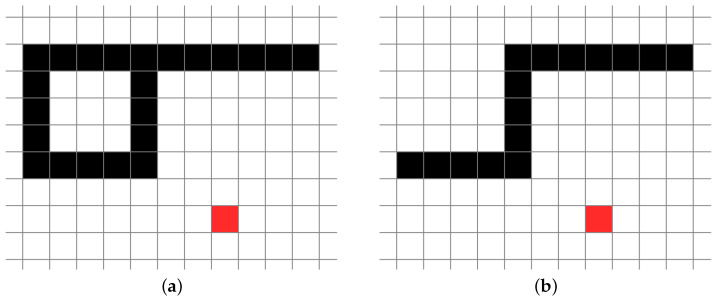
Example of the non-occluded pixels: (**a**) the black pixels are contour pixels in foreground lines, the white pixels are background pixels, and the red pixel denotes pixel *i* in the background pixels; (**b**) the non-occluded pixels in the contour pixels are exposed to the light from *i*, assuming that there is a light source at *i*.

In cognitive studies, the PPF represents the effect of shape contours on psychological intensities [6]. The computational PPF theory was only established for two-dimensional binary images [17,18]. To construct the PPF, the potential value, $p_{i}$, of the background pixel *i* is calculated as follows:

(1) $$p_{i}=\frac{1}{n}\sum_{k=1}^{n}\frac{1}{d_{k}},$$

where *n* is the number of contour pixels of lines and *d* is the distance between *i* and each contour pixel. Note that the contour pixels are the only pixels that are not occluded from the other contour pixels. Specifically, if *i* is a light source, all pixels of the portion exposed to light are non-occluded [15], as shown in Figure 4.

Similarly to the RDF-FM similarity calculation, the line pixels were neglected in the similarity calculation because potential values could not be obtained for foreground pixels.

## 4. Results

**Figure 5:**
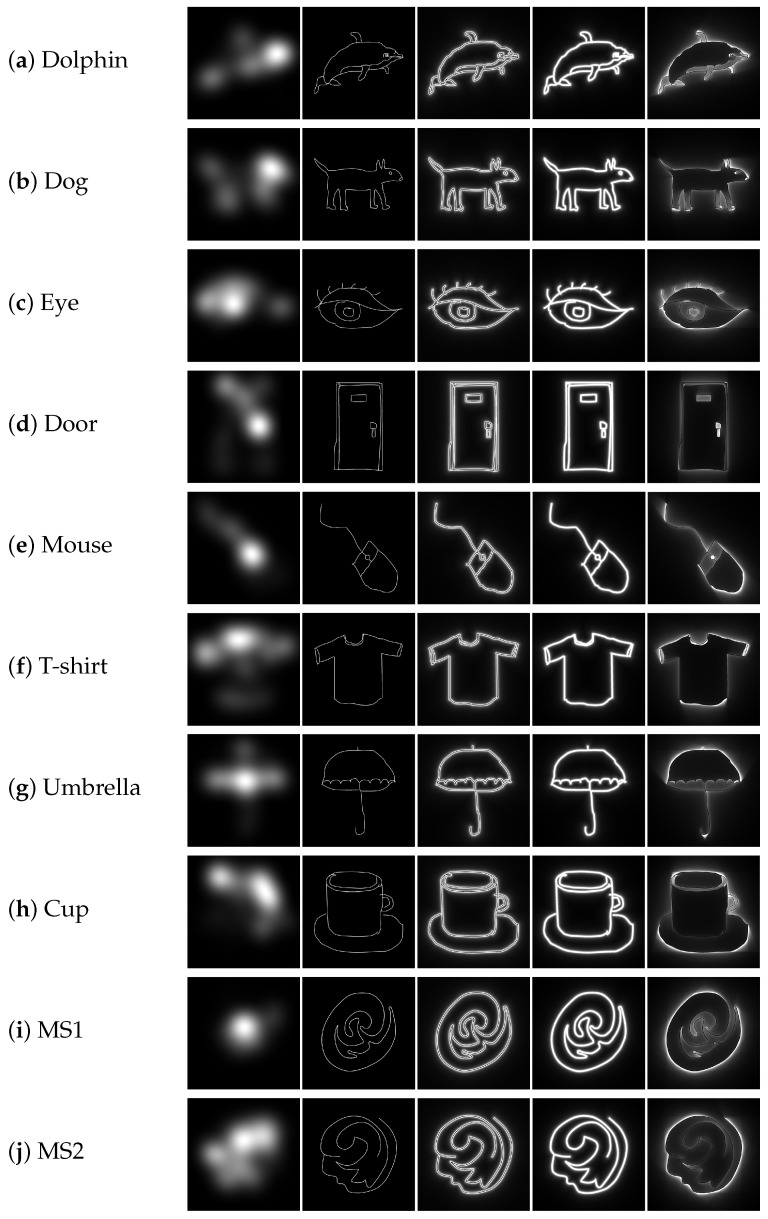
Results of the visualized features (from left to right): FM, BIN, RDF, BIN + RDF, and PPF.

Figure 5 shows the visualization results of the FMs and four image features, where white and black areas represent high and low values, respectively. The FMs and four image features are significantly different. In addition, each image feature captures different properties of the line-drawing objects. It is difficult to understand the visualization results because the values of the RDF, BIN + RDF, and PPF exponentially increase close to the shapes. Therefore, we multiplied α with the pixel values of the three features and set an upper limit of 1.0, where α=5 for the RDF and BIN + RDF, and α=20 for the PPF. This was applied only for visualization, as shown in Figure 5.

**Figure 6:**
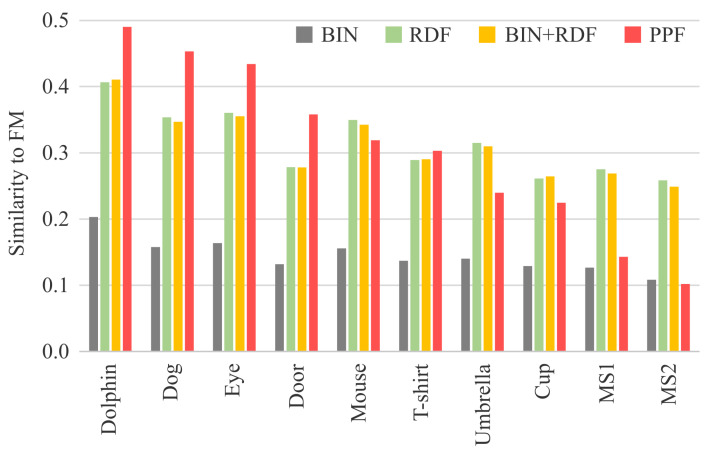
Graph of the similarity values in Table 1.

**Figure 7:**
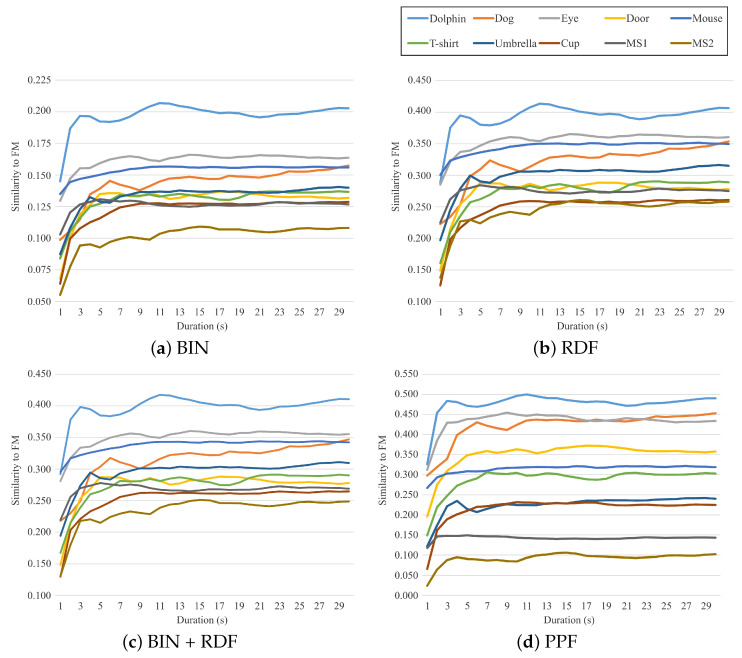
Similarity transitions during the viewing duration.

**Figure 8:**
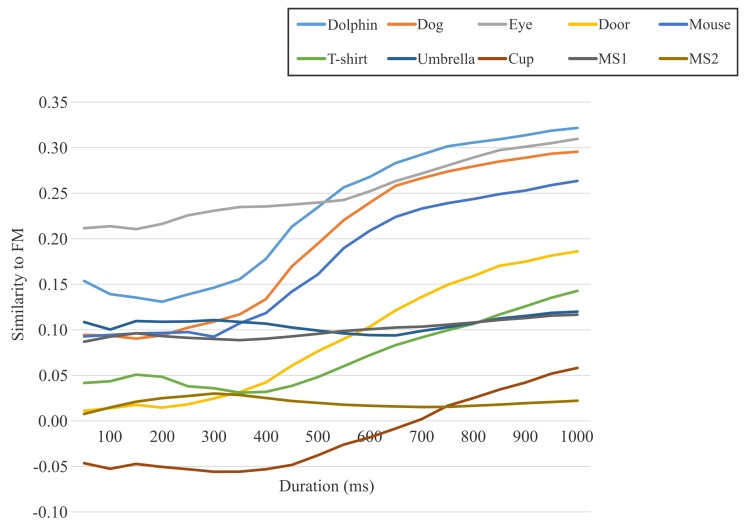
Similarity transitions within 1 s in PPF.

The degrees of similarity between the FM and four image features were difficult to establish through visual inspection but could be quantified by the similarity metric values. Table 1 and Figure 6 show the similarity results for the FMs obtained using all fixation points acquired for 30 s. Figure 7 shows the similarity transitions during 30 s. Each similarity value at 30 s corresponds to those in Table 1 and Figure 6. In Figure 7, the similarity values are low during the first few seconds because of relatively fewer fixation points. Table 1 and Figure 6 show the results after the stabilization of the fixation points. Furthermore, Figure 8 shows the similarity transitions from the start to 1 s in the PPF because animals and nonanimals can be unconsciously distinguished within 1 s [40].

As described in Section 3, the similarity metrics were based on the CC. We used a significance level of 0.01, and the *p*-values of the similarity metrics were sufficiently lower than 0.01. However, note that the general rule of thumb is not important; for example, the assumption of a strong correlation for values exceeding 0.7 does not hold. This is because we compared the FMs and distinctly different image features, which should be regarded as an image similarity rather than the CC. Another reason is that it is important to compare the relative values of similarity metrics rather than their magnitudes.

## 5. Discussion

The portions with a few changes or complexities are mainly viewed, as shown in the FMs in Figure 5. However, it was difficult to determine a trend for meaningless shapes. The center was preferentially viewed in MS1, whereas broad areas were viewed in MS2. Additional research with more meaningless shape stimuli is required to understand the trends and factors that affect eye fixations. The visualization results showed that the four image features differed depending on the shapes, indicating that they captured different properties of the shapes. Although the RDF and PPF were based on the distances between pixels, their visualization results significantly differed.

As shown in Table 1 and Figure 6, the similarity values between BIN and FM for the animate object stimuli were slightly larger than those for the other stimuli. In addition, the similarity values for the inanimate object and meaningless shape stimuli were almost identical. Specifically, when we conducted tests for the significance of the difference between a pair of CCs (similarities) [41], the *p*-values for Dog and Mouse (0.249), T-shirt and Umbrella (0.012), and Cup and MS1 (0.144) were larger than the significance level. Furthermore, the range of the similarity values was narrow, with a standard deviation (SD) of 0.02. Therefore, it was difficult to distinguish between the object categories using the BIN feature.

The similarity values between RDF and FM decreased from animate to inanimate objects to meaningless shapes. However, the values were almost identical for Door and MS1, and the value for MS1 was higher than that for Cup. In addition, the *p*-value for Cup and MS2 (0.019) was more than the significance level. They indicated the difficulty in distinguishing inanimate and meaningless shapes using the RDF feature, although the range of the similarity values was wider than that of the BIN, with an SD of 0.05. The similarity values between BIN + RDF and FM were similar to those of the RDF and FM, and the *p*-values were lower than the significance level. This indicates the effectiveness of considering line pixels in the similarity evaluation; the effectiveness could be increased if line drawings consisted of more complicated lines. However, as the similarity values between BIN + RDF and FM were similar to those of the RDF and FM, the trends remained similar; for example, the values were almost identical for Door and MS1 and the value for MS1 was higher than that for Cup. Therefore, it was difficult to distinguish inanimate and meaningless shapes using the BIN + RDF feature.

The similarity values between PPF and FM were the highest for animate objects, followed by inanimate objects, and meaningless shapes, and these values exhibited the widest range (SD = 0.12). In general, even if the value range is narrow, it would not be an issue if we can relatively differentiate values based on the object category. However, this was a relatively desirable result that had a more distinct trend than the trend for values of other image features. Furthermore, it should be noted that all *p*-values were lower than the significance level. In addition, the difference in similarity values was the largest between Cup and MS1, followed by the difference between the Eye and Door. These can be considered as boundaries for differentiating between inanimate objects and meaningless shapes and between animate and inanimate objects. Specifically, we can easily set thresholds to distinguish the object categories, such as animate:inanimate = 0.4 and inanimate:meaningless = 0.2.

Figure 7 indicates the manner in which the similarity values between each image feature and FM changed during the duration of viewing. They increased during the first few seconds and then stabilized. Although we requested that the participants view the stimuli for 30 s, this result indicated that it was enough to view the stimuli for 5–10 s. Simultaneously, this suggested that the participants had viewed most parts required for recognition during the first few seconds. These results are consistent with the result that the categorical object information was distinguished at an early stage of human perception [42]. In the first few seconds, the similarity values of the BIN, RDF, and BIN + RDF to the FMs did not always correctly categorize inanimate objects and meaningless shapes, as illustrated in Figure 7a–c. These values were quite complicated and could not be distinguished by the object category. In contrast, the similarity values between PPF and FM easily distinguished the object categories, as illustrated in Figure 7d. Although the similarity value for MS1 was higher than that for Cup at 1 s, they were subsequently reversed. Therefore, the PPF-FM similarity could be better categorized between 2 and 30 s.

Figure 8 shows the PPF-FM similarity transitions within 1 s. The similarity values stabilized until approximately 350 ms and increased thereafter. Three important findings have already been reported in relation to this result. First, perceiving animals and nonanimals caused a distinct difference within 150 ms of event-related potentials (ERPs) [43]. Second, ERPs showed a second component that was correlated with object recognition from 150 to 300 ms [44]. Third, the typical fixation duration was 150–300 ms [45]. These findings indicated that objects were categorized around the first fixation point. The stabilized values until approximately 350 ms in Figure 8 indicate that the earlier fixation points did not move around the first viewing point. Moreover, these values could not be categorized into object categories. Therefore, a real-time assessment of object perception using the PPF-FM similarity was impossible. This was also clear from the fact that the FMs were the convolutional images of fixation points and their changes appeared to be statistically delayed. Nevertheless, as shown in Figure 7d, the PPF-FM similarity could be better categorized after a few seconds. The similarity values increased from 350 ms in Figure 8 to 3 s in Figure 7d. This could be interpreted as the similarity values indicate a degree for some recognition processes as to whether perceived objects are correct. For instance, if you see a dog, the values confirm whether it is a dog. If this is true, the final similarity values may indicate a relative degree of cognition.

This study has certain limitations. The results cannot be generalized because we only used a few stimulus images. Moreover, the results do not consider different generations or ethnic groups because all participants were Japanese university students. Therefore, large-scale experiments are required to generalize the findings of this study.

## 6. Conclusions

This study assessed the effectiveness of assessing the human perception of object categorization in line drawings using similarity between the PPF and FMs by comparing the features of binary line-drawing images. The FMs of ten stimuli were generated from tracked eye movement data. The four features of binary images (BIN, RDF, BIN + RDF, and PPF) were computed and compared to the FMs using the similarity based on the CC. Only PPF more clearly distinguished the three object categories when compared to the other image features.

In the future, investigations must be conducted with more types of object stimuli and participants from different generations or ethnic groups for a detailed assessment of object perception. Furthermore, our results indicate that the final similarity values may represent a degree of cognition. This must be clarified through additional experiments using meaningless shapes. Specifically, if meaningless shapes can be interpreted as unknown shapes, we can verify whether common and meaningless shapes can be distinguished by the PPF-FM similarity to determine whether known and unknown shapes can be assessed.

## Figures and Tables

**Table 1:** Similarity values between the FM and the BIN, RDF, BIN + RDF and PPF image features. All *p*-values were significantly lower than 0.01.

	Dolphin	Dog	Eye	Door	Mouse	T-Shirt	Umbrella	Cup	MS1	MS2
BIN	0.203	0.157	0.164	0.132	0.156	0.137	0.140	0.129	0.127	0.108
RDF	0.407	0.354	0.361	0.278	0.350	0.289	0.315	0.261	0.275	0.258
BIN + RDF	0.411	0.347	0.356	0.278	0.343	0.290	0.310	0.265	0.269	0.249
PPF	0.490	0.453	0.434	0.358	0.319	0.303	0.240	0.225	0.143	0.102

## Data Availability

The image dataset containing the animate and inanimate objects can be accessed at the following: https://saliency.tuebingen.ai/datasets.html (accessed on 31 January 2022).
